# Opposite effects of interleukin-4 and interleukin-10 on nitric oxide production in murine macrophages

**DOI:** 10.1155/S096293519600018X

**Published:** 1996-04

**Authors:** A. Maru, S. K. Jackson

**Affiliations:** Department of Medical Microbiology University of Wales College of Medicine Cardiff CF4 4XN UK

## Abstract

Interleukin-4 (IL-4) and interleukin-10 (IL-10) were evaluated for their ability to inhibit the production of nitric oxide (NO) by interferon-γ (IFN-γ)- or lipopolysaccharide (LPS)-activated murine macrophages (RAW 264.7 and J774.2). Macrophages pre-treated with IL-4 and then stimulated with IFN-γ or LPS showed significant inhibition in their ability to produce NO as measured by nitrite production. Simultaneous treatment of IL-4 pre-incubated cells with IFN-γ and LPS together augmented nitrite accumulation. On the other hand, similar exposures of the macrophages to IL-10 followed by IFN-γ or LPS treatments resulted in significantly increased NO production. Thus IL-10 failed to suppress IFN-γ or LPS-induced NO production and showed opposite effects in these experiments to IL-4. We conclude that the two lymphokines have differing roles in the control of production of NO and might act to control the secretion of nitric oxide *in vivo*.


Mediators of Inflammation 5, 110–112 (1996)

### Opposite effects of interleukin-4 and interleukin-10 on nitric oxide production in murine macrophages

A. Maru and S. K. Jackson[CA]

Department of Medical Microbiology, University of Wales College of Medicine, Cardiff CF4 4XN, UK. Fax: (+44) (0)1222 744123

[CA]Corresponding Author

**INTERLEUKIN-4 (IL-4) and interleukin-10 (IL-10) were evaluated for their ability to inhibit the production of nitric oxide (NO) by interferon-γ (IFN-γ)- or lipopolysaccharide (LPS)-activated murine macrophages (RAW 264.7 and J774.2). Macrophages pre-treated with IL-4 and then stimulated with IFN-γ or LPS showed significant inhibition in their ability to produce NO as measured by nitrite production. Simultaneous treatment of IL-4 pre-incubated cells with IFN-γ and LPS together augmented nitrite accumulation. On the other hand, similar exposures of the macrophages to IL-10 followed by IFN-γ or LPS treatments resulted in significantly increased NO production. Thus IL-10 failed to suppress IFN-γ or LPS-induced NO production and showed opposite effects in these experiments to IL-4. We conclude that the two lymphokines have differing roles in the control of production of NO and might act to control the secretion of nitric oxide *in vivo*.**

**Key words**: Griess reaction, interferon-γ, interleukin-4, interleukin-10, lipopolysaccharide, macrophages, nitric oxide, nitrite

### Introduction

Murine macrophage cell lines or peritoneal macrophages express high levels of inducible nitric oxide synthase (iNOS) and secrete large amounts of nitric oxide (NO) when activated with lipopolysaccharide (LPS) or cytokines.[1–3] Macrophage activation for the expression of iNOS and production of NO is a multisignal and multistep process and involves the L-arginine:NO pathway, where NO is synthesized from the guanidino nitrogen of L-arginine by the enzyme iNOS.[4–7]

A few purified cytokines are known to suppress macrophage nitric oxide induction *in vitro*. Transforming growth factor-β (TGF-β) and macrophage deactivation factor (MDF) are two previously described agents affecting lymphocyte function. Interleukin 10 (IL-10) and interleukin 4 (IL-4), secretory products of $Th_2$ helper T lymphocytes ($Th_2$ cells) are cytokines whose biological actions antagonize IFN-γ secreting $Th_1$ helper T lymphocytes ($Th_1$ cells).[8] The antagonistic effect of the two groups of cells is cross-modulatory in action and is thought to be a mechanism that plays a role in homeostasis.

According to a number of investigators, the treatment of macrophages by IL-10 and IL-4 suppresses the induction and production of TNF-α, reactive oxygen intermediates (ROI) and reactive nitrogen intermediates (RNI) that are antimicrobial and tumoricidal in action.[9–11] In this respect, $Th_1$ secretory products are thought to play a role in host-defence whereas $Th_2$ cells are disease-promoting.[12]

The purpose of the present study was to compare and contrast the *in vitro* IL-4 and IL-10 macrophage deactivation and inhibition of NO secretion in IFN-γ- and LPS- activated macrophage-like murine cell lines.

### Materials and Methods

*Cytokines and biochemicals:* Recombinant murine IFN-γ, IL-10 and IL-4 were purchased from Genzyme Diagnostics, Kent, UK. Lipopolysaccharide from *E. coli* O:111 B4, sulphanilamide, sodium nitrite, and *N*-(1-naphthyl)-ethylenediamine dihydrochloride were purchased from Sigma Chemical Co. Ltd, Poole, UK. Orthophosphoric acid was purchased from BDH Chem. Co. Ltd, Poole, UK. Foetal calf serum, L-glutamine and RPMI 1640 medium were purchased from Gibco (Life Technologies) Ltd, Paisley, UK.

*Cells and cell cultures:* Macrophage-like murine cell lines (RAW 264.7 and J774.2) were obtained from the European Collection of Animal Cell Cultures, Salisbury, Wiltshire, UK. The macrophages were cultured in RPMI 1640 medium supplemented with foetal calf serum (5%), L-glutamine (2 mM), streptomycin (100 g/ml) and penicillin (100 U/ml) for 3–4 days. Prior to experiments, the cells were washed and resus-

© 1996 Rapid Science Publishers pended in fresh medium and their number adjusted to 2 × $10^6$/ml medium. The cells were then plated into 96-well microtitre plates (5 × $10^5$ cells/well in a volume of 250 µl) in triplicate and incubated for 2 h at 37°C in a 5% $CO_2$ atmosphere to allow them to adhere. The supernatant was removed by aspiration and the cell monolayers were washed twice to remove non-adherent cells. Cell viability was always greater than 90% as determined by the trypan blue dye exclusion criterion.

*Addition of cytokines and lipopolysaccharide:* The washed cell monolayers were treated with IL-10 (100 U/ml) or IL-4 (100 U/ml) and incubated for 20 h prior to the addition of murine IFN-γ or LPS. Dose–response experiments and our previous work[13] and our unpublished observations showed that 50–100 U/ml of IL-4 and IL-10 was a minimum concentration to produce maximal effects in the current assays. Thus a concentration of 100 U/ml was used for these cytokines throughout. After incubation, the supernatant was aspirated and the cell monolayers were washed twice with medium. The cells were then re-exposed to the same concentrations of IL-4 and IL-10 in the presence of IFN-γ (100 U/ml) and/or LPS (100 ng/ml) for NO production. Cells were also treated with either IL-4, IL-10, IFN-γ or LPS alone for 20 h to act as controls.

*Nitrite assay:* After 20 h of incubation, the presence of nitrite in the culture medium as an indication of NO production was determined by the Griess reaction as described previously.[14] Briefly, supernatant (100 µl) from each well was mixed with an equal volume of Griess reagent [1 part of 0.1% *N*-(1-naphthyl)-ethylenediamine dihydrochloride in distilled water plus 1 part of 1% sulphanilamide in 5% concentrated phosphoric acid] in a 96-well microtitre plate and incubated at room temperature in the dark for 10–20 min. The optical density of the chromogen was measured at 540 nm with a microtitre plate reader (Titertek multiscan). Nitrite concentration in the supernatant was determined in nanomoles from a standard curve generated with sodium nitrite ranging from 1 to 200 nmol.

*Calculation and statistical analysis:* Nitrite concentrations for each cell group were calculated from the standard curve and data are expressed as nmol of nitrite per 5 × $10^5$ cells. All values represent the mean and standard error (S.E.) of three separate experiments performed in triplicate. Statistical analysis was performed with the Student *t*-test, using Minitab Release 8.2 software.

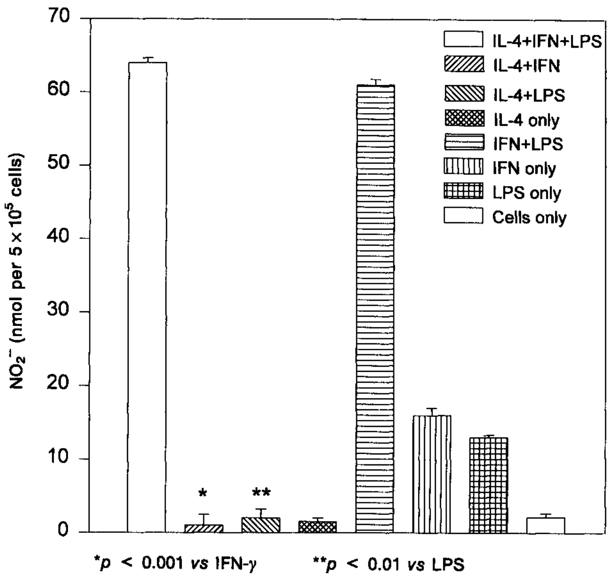

FIG. 1. Effect of IL-4 (100 U/ml) pre-treatment on IFN-γ (IFN)- (100 U/ml) or LPS- (100 ng/ml) stimulated nitric oxide production in RAW 264.7 macrophages. Results shown are mean ± S.E. for three separate experiments performed in triplicate.

### Results

When RAW 264.7 macrophages were activated with either murine IFN-γ or LPS, or IFN-γ plus LPS, high levels of nitrite were measured in the culture supernatants assayed. Pre-incubation of the same macrophages with IL-4 for 20 h and treating them with either IFN-γ or LPS inhibited NO production (Fig. 1). Inhibition difference was observed to be highly significant between results of IFN-γ plus IL-4 and IFN-γ alone ($p < 0.001$) and LPS plus IL-4 and LPS only ($p < 0.01$). Treatment of macrophages with IL-4 only for 20 h or more failed to induce NO production when compared with the controls (untreated cells). Furthermore, simultaneous treatment of IL-4 pre-treated cells with IFN-γ in the presence of LPS could not inhibit the production of NO (Fig. 1). This result suggests that IL-4 is able to inhibit or block either LPS or IFN-γ-induced NO production but is unable to overcome the combined effect of these stimuli. The same pattern of results was recorded when a different cell line (J774.2) was used (data not shown).

On the other hand, when the RAW 264.7 cells were pre-incubated with IL-10 for 20 h and then stimulated with either IFN-γ or LPS or with both agents, there was no inhibition of nitrite production compared with controls (Fig. 2). Indeed, IL-10 pre-treatment was found to significantly increase the production of nitric oxide in cells stimulated with IFN-γ ($p < 0.01$) or LPS ($p < 0.05$). Besides, even at higher concentrations (500 U/ml) or increased or decreased incubation times, IL-10 was not capable of suppressing NO

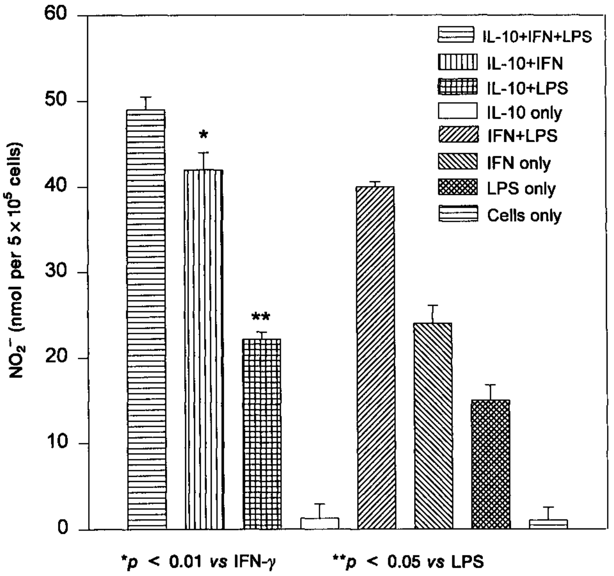

FIG. 2. Effect of pre-treatment of macrophages (RAW 264.7) with IL-10 (100 U/ml) on IFN-γ (100 U/ml) or LPS- (100 ng/ml) induced nitric oxide production. Data are mean ± S.E. for three separate experiments performed in triplicate.

production (results not shown). Furthermore, Fig. 2 shows that IL-10 in the absence of IFN-γ or LPS had no effect on the induction of NO in the macrophages used. Interleukin-10 was found to have the same nitric oxide potentiating effects in J774.2 cell lines (data not shown).

### Discussion

This study demonstrates the clear differences between the actions of the $Th_2$ lymphokines, IL-10 and IL-4 on the LPS- or IFN-γ-stimulated production of nitric oxide in murine macrophages. The suppression of nitric oxide synthesis by pre-incubation of the cells with IL-4 is in agreement with the work of other investigators.[8,10,15,16]

According to Bogdan and colleagues,[17] IL-10 was found to be a weak inhibitor of reactive nitrogen intermediates production in activated macrophages. Contrary to the observation of a number of other investigators,[9–12] IL-10 failed to inhibit IFN-γ- or LPS-induced NO production in both murine macrophage cell lines examined in this work. Moreover, in this study we found that IL-10 could enhance the LPS- or IFN-γ-stimulated production of nitric oxide. In this respect our results are in agreement with the work of other investigators[18,19] who also reported that IL-10 could enhance nitric oxide production.

Recently, it has been suggested that at least some of the effects of lymphokines on macrophages may be mediated via changes in lipid metabolism.[13] In this regard, it was found that IL-4, IL-10 and IFN-γ had different effects on macrophage fatty acid metabolism, and this was correlated with different effects on TNF-α and nitric oxide production.[20]

In conclusion, although the two subsets of lymphocytes ($Th_1$ and $TH_2$), with distinct biological functions, are known to have antagonistic roles, our data demonstrate that $Th_2$ cytokine products (IL-4 and IL-10) have different effects on IFN-γ- or LPS-induced NO production in macrophages. It is possible that these lymphokines regulate the production of NO *in vivo* so that maximal bactericidal and minimal host tissue destruction are elicited. The molecular basis for the differing roles of lymphokines in the control of NO production is currently under investigation in our laboratory.

### References

1. Stuehr DJ, Marletta MA. Synthesis of nitrite and nitrate in murine macrophage cell lines. *Cancer Res* 1987; **47**: 5590–5594.
2. Ding AH, Nathan CF, Stuehr DJ. Release of reactive nitrogen intermediates and reactive oxygen intermediates from mouse peritoneal macrophages: comparison of activating cytokines and evidence for independent production. *J Immunol* 1988; **141(7)**: 2407–2412.
3. Nussler AK, Billiar TRJ. Inflammation, immunoregulation and inducible nitric oxide. *Leukoc Biol* 1993; **54**: 171–178.
4. Tayeh MA, Marletta MA. Macrophage oxidation of L-arginine to nitric oxide, nitrite and nitrate. *J Biol Chem* 1989; **264(3)**: 19654–19658.
5. Moncada S, Higgs EA, Hodson HF, *et al.* The L-arginine:nitric oxide pathway. *J Cardiovasc Pharmacol* 1991; **17(Suppl.3)**: S1–S9.
6. Moncada S, Palmer RMJ, Higgs EA. Nitric oxide: physiology, pathophysiology and pharmacology. *Pharmacol Rev* 1991; **43(2)**: 109–142.
7. Green SJ, Nancy CA. Antimicrobial and immunopathologic effects of cytokine-induced nitric oxide synthesis. *Curr Opin Infect Dis* 1993; **6**: 384–396.
8. Liew FY, Li Y, Severn A, Millots S, Schmidt J, Salter M, Moncada S. A possible pathway of regulation by murine T helper type-2 ($Th_2$) cells of a $Th_1$ cell activity via the modulation of the induction of nitric oxide synthase on macrophages. *Eur J Immunol* 1991; **21(10)**: 2489–2494.
9. Moore KW, O'Garra A, Malefyt RW, Vieira P, Mosmann TR. Interleukin-10. *Annu Rev Immunol* 1993; **11**: 165–190.
10. Cenci E, Romani L, Mencacci A, Spacca-Schiaffella E, Puccetti P, Bistoni F. Interleukin-4 and interleukin-10 inhibit nitric oxide-dependent macrophage killing of *Candida albicans*. *Eur J Immunol* 1993; **23**: 1034–1038.
11. Cunha FQ, Moncada S, Liew FY. Interleukin-10 (IL-10) inhibits the induction of nitric oxide synthase by interferon-γ in murine macrophages. *Biochem Biophys Res Commun* 1992; **182(3)**: 1155–1159.
12. Fiorentino DF, Zlotnik A, Mosmann TR, Howard M, O'Garra A. IL-10 inhibits cytokine production by activated murine macrophages. *J Immunol* 1991; **147(11)**: 3815–3822.
13. Darmani H, Harwood JL, Jackson SK. Interferon-γ-stimulated uptake and turnover of linoleate and arachidonate in macrophages: a possible pathway for hypersensitivity to endotoxin. *Cell Immunol* 1993; **152**: 59–71.
14. Green L, Wagner DA, Glogowski J, Skipper PL, Wishnok JH, Tannenbaum SR. Analysis of nitrate, nitrite and [$N^{15}$]nitrite in biological fluids. *Anal Biochem* 1982; **126**: 131–138.
15. Al-Ramadi BK, Meissler Jr JJ, Huang D, Eisenstein TK. Immunosuppression by nitric oxide and its inhibition by interleukin-4. *Eur J Immunol* 1992; **22**: 2249–2254.
16. Bogdan C, Vodovotz Y, Paik J, Xie Q-W, Nathan C. Mechanism of suppression of nitric oxide synthase expression by interleukin-4 in primary mouse macrophages. *J Leukoc Biol* 1994; **55**: 227–233.
17. Bogdan C, Vodovotz Y, Nathan C. Macrophage deactivation by interleukin-10. *J Exp Med* 1991; **174**: 1549–1555.
18. Corradin SB, Fasel N, Buchmuller-Rouiller Y, Ransijn A, Smith J, Mauel J. Induction of macrophage nitric oxide production by interferon-γ and tumour necrosis-α is enhanced by interleukin-10. *Eur J Immunol* 1993; **23**: 2045–2048.
19. Chesrown SE, Monier J, Visner G, Nick HS. Regulation of nitric oxide synthase mRNA levels by LPS, IFN-γ, TNF-β and IL-10 in murine macrophage cell lines and rat peritoneal macrophages. *Biochem Biophys Res Commun* 1994; **200(1)**: 126–134.
20. Darmani H, Harwood HL, Parton J, Jackson SK. Macrophage activation by lipopolysaccharide, interferon-γ and interleukin-4: effect of fatty acid metabolism. *Mediators Inflam* 1994; **4(1)**: 25–30.

**Received 25 November 1995; accepted 10 January 1996**
